# Clinical outcomes after first‐line HIV treatment failure in South Africa: the next cascade of care

**DOI:** 10.1111/hiv.12877

**Published:** 2020-06-03

**Authors:** CC Iwuji, M Shahmanesh, O Koole, K Herbst, D Pillay, MJ Siedner, K Baisley, the H‐DREAM Network

**Affiliations:** ^1^ Department of Global Health and Infection Brighton and Sussex Medical School Brighton UK; ^2^ Africa Health Research Institute KwaZulu-Natal South Africa; ^3^ Research Department of Infection & Population Health University College London London UK; ^4^ London School of Hygiene and Tropical Medicine London UK; ^5^ SAPRIN South African Medical Research Council Cape Town South Africa; ^6^ Division of Infection and Immunity University College London London UK; ^7^ Harvard Medical School Boston MA USA

**Keywords:** antiretroviral therapy, HIV drug resistance, South Africa, viral load monitoring, virological failure

## Abstract

**Introduction:**

There is limited literature on the appropriateness of viral load (VL) monitoring and management of detectable VL in public health settings in rural South Africa.

**Methods:**

We analysed data captured in the electronic patient register from HIV‐positive patients ≥ 15 years old initiating antiretroviral therapy (ART) in 17 public sector clinics in rural KwaZulu‐Natal, during 2010–2016. We estimated the completion rate for VL monitoring at 6, 12, and 24 months. We described the cascade of care for those with any VL measurement ≥ 1000 HIV‐1 RNA copies/mL after ≥ 20 weeks on ART, including the following proportions: (1) repeat VL within 6 months; (2) re‐suppressed; (3) switched to second‐line regimen.

**Results:**

There were 29 384 individuals who initiated ART during the period [69% female, median age 31 years (interquartile range 25–39)]. Of those in care at 6, 12, and 24 months, 40.7% (9861/24 199), 34% (7765/22 807), and 25.5% (4334/16 965) had a VL test at each recommended time‐point, respectively. The VL results were documented at all recommended time‐points for 12% (2730/22 807) and 6.2% (1054/16 965) of ART‐treated patients for 12 and 24 months, respectively. Only 391 (18.3%) of 2135 individuals with VL ≥ 1000 copies/mL on first‐line ART had a repeat VL documenting re‐suppression or were appropriately changed to second‐line with persistent failure. Completion of the treatment failure cascade occurred a median of 338 days after failure was detected.

**Conclusion:**

We found suboptimal VL monitoring and poor responses to virologic failure in public‐sector ART clinics in rural South Arica. Implications include increased likelihood of morbidity and transmission of drug‐resistant HIV.

## Introduction

UNAIDS estimates that to end HIV/AIDS as a public health threat by 2030, 95% of people living with HIV must be diagnosed, 95% of those diagnosed need to be taking antiretroviral therapy (ART), and 95% of those must achieve long‐term virologic suppression [1]. To ascertain this third target, HIV treatment programmes in sub‐Saharan Africa (SSA) will need to scale up viral load (VL) monitoring, and to institute prompt and appropriate management strategies for those failing therapy.

According to WHO guidelines, patients should receive VL monitoring at months 6 and 12 after ART initiation, and then annually thereafter. Those with a VL ≥ 1000 HIV‐1 RNA copies/mL should be retested after 3–6 months of adherence counselling support, and then either retained on first‐line therapy if re‐suppressed or switched to second‐line therapy if VL ≥ 1000 copies/mL [2]. Yet, despite the rapid and successful scaling‐up of VL monitoring, little is known about how these VL guidelines are being used in clinical decision‐making in public ART programmes in SSA. With increases in drug resistance in the region [3, 4, 5], there is concern over an epidemic of ART failure compromising already challenging efforts to combat HIV/AIDS as a public health threat.

We observed HIV‐positive individuals after ART initiation in a programmatic clinical cohort in KwaZulu‐Natal, to determine whether guidelines about management of virologic failure are being implemented. We hypothesized that poor adherence to VL monitoring guidelines has resulted in poor identification of treatment failure and prolonged duration on failing first‐line regimens.

## Methods

### Setting, study design and population

This study was conducted in the Hlabisa sub‐district as part of the Africa Health Research Institute (AHRI) demographic surveillance and clinical population cohort [6]. All HIV‐positive individuals who initiate ART at any of the 17 area‐based public sector clinics have had clinical data prospectively recorded through the Three Interlinked Electronic Register (TIER.net) since 2013. Prior to this period, data were back‐captured in TIER.net. All data were linked to the AHRI longitudinal population cohort. At the time of linkage, visits with implausible dates (VL test dates, date of ART initiation) and duplicate visits resulting from back capturing when a patient is transferred from one clinic to another were dropped. ART regimen codes were also examined for systematic errors. In this analysis, we included all individuals during 2010–2016 in care at a public‐sector HIV clinic with a recorded ART initiation date.

### Viral load monitoring

Since 2010, South African HIV treatment guidelines have recommended VL monitoring in all HIV‐positive individuals at 6 and 12 months after initiating ART, and 12‐monthly thereafter if virally suppressed [7]. HIV‐positive individuals attending the healthcare clinics undergo venepuncture by the clinic staff with plasma samples transported to the Hlabisa District hospital daily, where HIV‐1 VL is done (*m*2000 RealTime System; Abbott Molecular, Abbott Park, Illinois, U.S.A)**.** The results are delivered to the clinics during sample pick‐up and are manually entered by clinic clerks into TIER.Net. The paper results are subsequently filed in patients' clinical charts. Patients who have an HIV VL ≥ 1000 copies/mL after at least 6 months on ART are recommended to receive adherence counselling followed by a repeat VL 2–3 months after the initial detection of elevated VL. If the repeat HIV VL is ≥ 1000 copies/mL, individuals are meant to be switched to second‐line ART. Those with VL < 1000 copies/mL remain on first‐line therapy.

### Statistical methods

Data were extracted from Tier.Net, containing HIV records for patients on ART in the sub‐district and analysed using Stata version 14 (College Station, TX, USA). We first estimated the completion rate for VL monitoring at 6, 12, and 24 months, by using VL measurements which fall within the 6‐month (≥ 5 and ≤ 9 months), 12‐month (> 9 and ≤ 15 months) and 24‐month (> 21 and ≤ 27 months) windows after ART initiation, respectively. The denominators for each of these time‐points were people who had started ART and were in care at the evaluated time point, although there might have been interruptions outside of the time periods evaluated. As a sensitivity analysis, we examined VL monitoring restricted to individuals who remained continuously in care over each time period.

We estimated optimal VL monitoring defined as completing both 6‐ and 12‐month VL for those on ART for a minimum of 15 months and at 6, 12, and 24 months for those on ART for a minimum of 27 months.

We described the cascade of care for all individuals with a VL measurement ≥ 1000 copies/mL after at least 5 months on ART, including the proportion with a repeat VL within 6 months (allow a window of 10–30 weeks after VL ≥ 1000 copies/mL), the proportion who re‐suppressed, and the proportion who changed to a second‐line regimen if a repeat VL remained ≥ 1000 copies/mL. For the cascade of care analysis, we restricted our analysis to the first episode of virologic failure for each individual in the dataset. We examined factors associated with successful management of the first‐line cascade using logistic regression, among all individuals with virologic failure. Successful management was defined as having a repeat VL test within 6 months, and either VL < 1000 copies/mL on repeat testing or switch to an appropriate second‐line therapy.

### Ethics

Ethical approval for the AHRI Population Intervention Platform Study and linkage to government ART records (TIER.net) was granted by the Biomedical Research Ethics Committee of the University of KwaZulu‐Natal, SA (BE290/16).

## Results

Between January 2010 and December 2016, 29 384 HIV‐positive individuals initiated ART in the public sector in our study catchment area (Table 1). The median age was 31 years [interquartile range (IQR): 26–39] and 69.9% were female. Of those on ART for 6, 12 and 24 months, we found that 40.7% (9861/24 199), 34% (7765/22 807), and 25.5% (4334/16 965) had a VL test documented at each recommended time‐point, respectively.

**Table 1:** Proportion of individuals aged ≥ 15 years with viral load (VL) monitoring, and with optimal monitoring, during first 12 and 24 months on antiretroviral therapy (ART)

Year of ART initiation	*N* starting ART	Median (IQR) age (years)	Female (%)	*n* with VL at 6 months/*N* on ART^†^ (%)	*n* with VL at 12 months/*N* on ART^‡^ (%)	*n* with VL at 24 months/*N* on ART^§^ (%)	*N* (%) with optimal monitoring to 12 months^¶^	*N* (%) with optimal monitoring to 24 months^#^
2010	3177	33 (27–41)	67.5	1234/2495 (49.5%)	869/2354 (36.9%)	839/2150 (39.0%)	361 (15.3%)	217 (10.1%)
2011	3543	34 (27–41)	69.4	1544/2961 (52.1%)	1021/2823 (36.2%)	764/2597 (29.4%)	381 (13.5%)	153 (5.9%)
2012	4040	33 (27–41)	66.7	1495/3406 (43.9%)	977/3235 (30.2%)	865/2993 (28.9%)	328 (10.1%)	146 (4.9%)
2013	3642	30 (25–38)	71.1	907/2897 (31.3%)	757/2741 (27.6%)	750/2523 (29.7%)	207 (7.6%)	101 (4.0%)
2014	3988	29 (24–37)	73.3	793/3231 (24.5%)	1017/3045 (33.4%)	619/2740 (22.6%)	252 (8.3%)	87 (3.2%)
2015	5556	31 (25–38)	70.6	2035/4695 (43.3%)	1391/4428 (31.4%)	497/3962 (12.5%)	519 (11.7%)	88 (2.2%)
2016	5438	31 (25–38)	69.8	1853/4514 (41.1%)	1733/4181 (41.4%)	–	682 (16.3%)	–
Total	29 384	31 (26–39)	69.9	9861/24 199 (40.7%)	7765/22 807 (34.0%)	4334/16 965 (25.5%)	2730 (12.0%)	1054 (6.2%)

^†^ Viral load measurement done between > 20 weeks and 9 months after starting ART. Denominator refers to individuals who have been on ART for at least 9 months.

^‡^ Viral load measurement done between > 9 and 15 months after starting ART. Denominator refers to individuals who have been on ART for at least 15 months.

^§^ Viral load measurement done between > 21 and 27 months after starting ART. Denominator refers to individuals who have been on ART for at least 27 months.

^¶^ Optimal monitoring during first 12 months is defined as a viral load measurement at 6 and 12 months after ART initiation, using the windows in footnotes † is > 20 weeks and 9 months and ‡ is > 9 months and 15 months. Denominator refers to individuals who have been on ART at least 15 months.

^#^ Optimal monitoring during the first 24m is defined as a viral load measurement at 6m, 12m and 24m after ART initiation, using windows in footnotes † is > 20 weeks and 9 months, ‡ is > 9 months and 15 months and § is > 21 months and 27 months. Denominator is individuals who have been on ART at least 27 months.

**Fig. 1:**
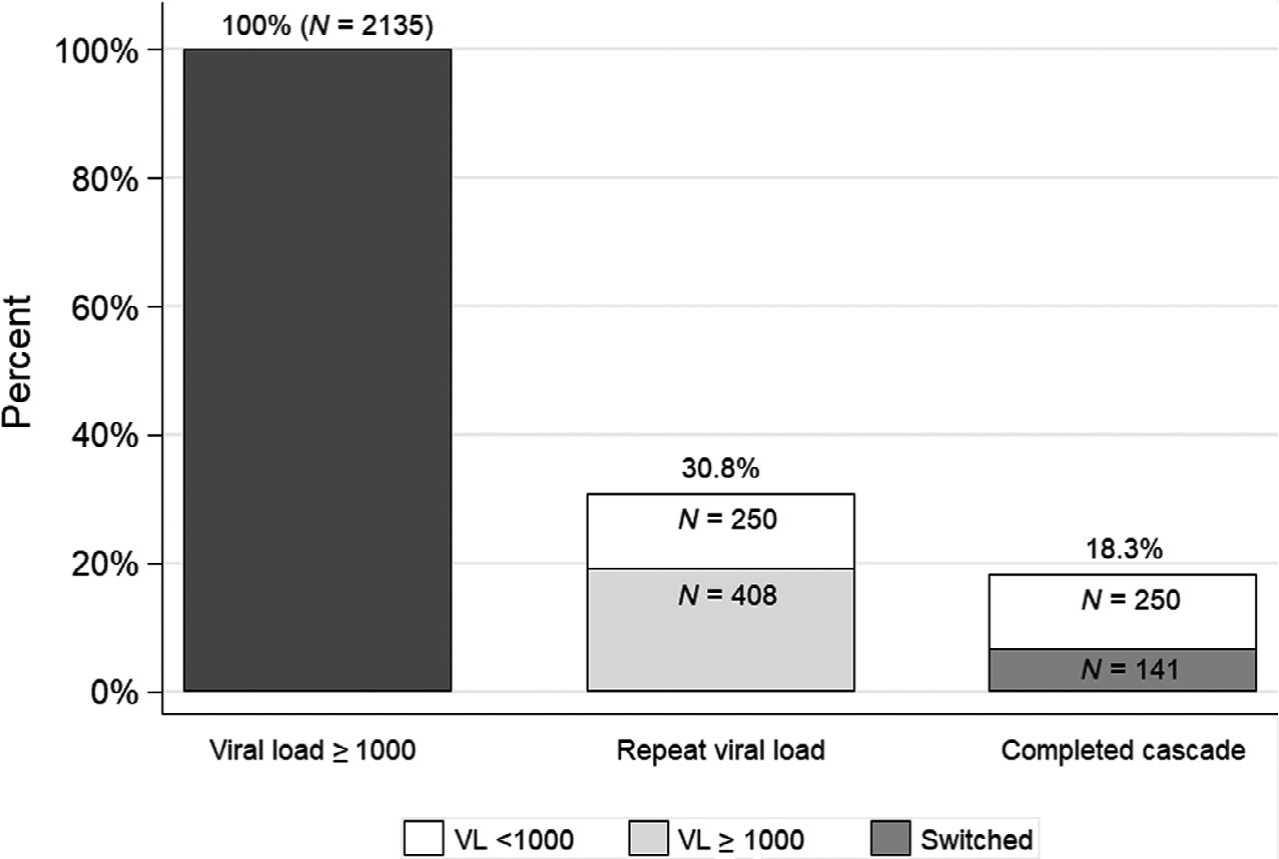
Cascade of care after detection of virological failure. VL, viral load.

Optimal VL monitoring including all recommended time‐points for those on ART for 12 and 24 months was documented for 12% (2730/22 807/22 730) and 6.2% (1054/16 965), respectively. The proportion of individuals with month‐24 VL monitoring recorded declined from 39.0% in 2010 to 12.5% in 2015. A similar decline was seen when optimal monitoring at 24 months was evaluated, going from 10.1% to 2.2%. Viral monitoring varied by clinic, with optimal monitoring at 12 months ranging from 2.7% to 33.9%, and that at 24 months ranging from 0.5% to 17.9%. (Table S1). The results of sensitivity analysis restricted to those continuously in care were similar to the main analysis (Table S2). Over the study period, 77.6% and 70.8% remained in care at 12 and 24 months after ART initiation, respectively (Table S3). Of the 29 384 individuals who started ART, 19 582 (66.6%) had at least one VL measurement recorded at any point > 20 weeks after starting ART. Ten per cent (2135) had at least one VL ≥ 1000 copies/mL (Table S4); 64.5% were female, and median (IQR) time to failure was 469 (264–849) days. Of those with VL ≥ 1000 copies/mL, 658 (30.8%) had a repeat VL recorded within 6 months, and 391 (18.3%) achieved successful management of virologic failure with either a repeat VL < 1000 copies/mL or appropriate change to second‐line therapy (Fig. 1). For the 141 individuals who switched to second‐line therapy, the median (IQR) time to regimen change was 338 days (135–713) after their first elevated VL measurement. In the multivariable analysis, successful management was strongly associated with increasing duration on ART and weakly associated with being female [1.23, 95% confidence interval (CI): 0.95–1.60, *P* = 0.11] and under 25 years of age (1.34, 95% CI: 0.99–1.83, *P* = 0.14) (Table S5).

## Discussion

In a representative population‐based longitudinal clinical cohort attending public sector primary care clinics in rural South Africa, we found significant deficits in VL monitoring and delayed or absent responses to virologic failure. Indeed, less than one in five individuals in this programmatic cohort were confirmed to have virologic re‐suppression or change to second‐line therapy after virologic failure, and those that did change therapy did so a median of 1 year after virologic failure. Such delays are likely to have significant deleterious individual and public health impacts through effects on patient morbidity, accumulation of drug resistance, and persistent risk of HIV transmission. Furthermore, suboptimal VL monitoring could lead to inaccurate decisions by policymakers on programme effectiveness and needs for second‐line ART.

There are many potential reasons for the poor VL monitoring observed in this treatment programme: patients not being aware of or empowered to request recommended blood tests, facility staff not having training, sufficient time or resources to conduct blood tests when due, failure of results to reach clinics from the laboratory or failure to capture results on TIER.Net. It is a limitation of this study that we did not review clinical records as our ethics permission did not cover this, hence we are uncertain which of these factors might account for this poor VL monitoring. The decline in optimal VL monitoring over time was most apparent for the 24 month time‐point; by contrast, there appeared to be some improvement in more recent years in optimal monitoring at 12 months. Hence, we speculate that this could be due to prioritization of monitoring of patients with advanced disease as they start care over the first few months of treatment and a relaxation in monitoring frequency as patients become more established on ART, especially in the context of high patient burden and staff shortages. If VL data in the TIER.net database are not missing at random, such that they are more likely to be missing among those with a suppressed VL, it would falsely bias our findings to suggest worse VL suppression rates. However, TIER.net is the system most often used for regional and national HIV reporting, so our data would be similar to what would be expected for programmatic reporting by the Department of Health. A study in Khayelitsha, South Africa, comparing laboratory VL data with clinic level VL database over a 12‐month period reported a higher VL completion (84%) with laboratory records but with only 55% captured in clinic database [8]. However, a South African study including data from four provinces examined a random sample of patients’ clinical records to ascertain the reason for missing VL and concluded that VL were missing on TIER.Net because they were not done [9]. Another clinical chart audit from 11 clinics in KwaZulu‐Natal demonstrated that only 42%, 32% and 26% of adult patients underwent VL testing at 6 months, 1 year and 2 years after ART initiation, respectively, which is similar to our findings [10]. These studies did not assess the use of VL results for clinical decision‐making.

But perhaps more concerning than suboptimal VL monitoring is the incredibly low response to detectable VL we observed. Our findings are similar to a Mozambican study which also reported a poor cascade of care in response to virologic failure: an initial VL test occurred on time in only 40% (17 236) of individuals, 18% of whom had an elevated first VL, and 35% of whom had an appropriate repeat. Amongst these 1095 with a follow‐up VL, 62% had virological failure. Of those, only a third started second‐line ART [11]. An assessment of the public ART programme in Lesotho following the roll‐out of VL monitoring in one district reported a similar poor health system response to detectable VL [12]. However, better management of detectable VL was reported in another Lesotho study within the context of a prospective study [13] as well as in Swaziland [14]. A South African modelling study investigated the reason for the high rates of nonnucleoside reverse transcriptase inhibitor (NNRTI) resistance in South Africa. The model was able to reproduce the time trends of HIV in South Africa from 2005 to 2016 and appropriately captured the dynamics of the spread of NNRTI resistance. The model demonstrated that the rapid scaling‐up of ART and inadequate switching to second‐line ART were the key drivers of the spread of NNRTI resistance in South Africa [15].

These data reinforce the urgent priority for ART care programmes across the region to identify strategies to ensure virologic failure is appropriately managed. Some proposed strategies include task‐shifting to allow nurses to change individuals to second‐line therapy with physician approval [16], immediate change to second‐line therapy after a first episode of virologic failure, use of point‐of‐care HIV VL testing to support immediate decisions by front‐line clinic staff [17], or incorporation of resistance testing to better differentiate between adherence and resistance‐based failure and encourage clinicians to respond appropriately [18]. The early detection and prompt management of virological failure will be a critical step towards achieving UNAIDS 95‐95‐95 targets by 2030 [1]**.**

## Author contributions

CI, DP, MJS and KB designed and implemented the study. KB did the statistical analysis. CI wrote the initial draft of the manuscript. All authors contributed to the review, interpretation and presentation of the findings. All authors approved the final version of the manuscript for submission.

## Supporting information


**Table S1** Proportion of individuals aged ≥ 15 years with viral load monitoring, and with optimal monitoring, during first 12 and 24 months on ART, by clinic.^1^
**Table S2** Proportion of individuals aged ≥ 15 years with viral load monitoring, and with optimal monitoring, during first 12 and 24 months on ART, among those who have been continuously in care without interruption.
**Table S3** Proportion of individuals aged ≥ 15 years starting ART in each year, and still in care at 6, 12 and 24 months.
**Table S4** Individuals aged ≥ 15 years with viral load ≥ 1000, and proportion with repeat test in 6 or 12 months, among individuals starting ART in 2010–2016.
**Table S5** Factors associated with success in the first‐line cascade, among 2135 individuals aged ≥ 15 years with a viral load ≥ 1000 copies/mL.
