# Supplementary material for: Clinical outcomes after first‐line HIV treatment failure in South Africa: the next cascade of care
Source: HIV Med. 2020 Jun 3;21(7):457–62. doi: 10.1111/hiv.12877 (PMC7384088; doi:10.1111/hiv.12877)
Supplement: Supplementary file 1 — Table S1 Proportion of individuals aged ≥ 15 years with viral load monitoring, and with optimal monitoring, during first 12 and 24 months on ART, by clinic.1 Table S2 Proportion of individuals aged ≥ 15 years with viral load monitoring, and with optimal monitoring, during first 12 and 24 months on ART, among those who have been continuously in care without interruption. Table S3 Proportion of individuals aged ≥ 15 years starting ART in each year, and still in care at 6, 12 and 24 months. Table S4 Individuals aged ≥ 15 years with viral load ≥ 1000, and proportion with repeat test in 6 or 12 months, among individuals starting ART in 2010–2016. Table S5 Factors associated with success in the first‐line cascade, among 2135 individuals aged ≥ 15 years with a viral load ≥ 1000 copies/mL. [file HIV-21-457-s001.docx]

**Supplementary Table 1. Proportion of individuals aged ≥15 years with viral load monitoring, and with optimal monitoring, during first 12m and 24m on ART, by clinic^1^**

| **Clinic of ART initiation** | **N starting ART^1^** | **n with VL at 6m  / N on ART^2^ (%)** | **n with VL at 12m  / N on ART^3^ (%)** | **n with VL at 24m   / N on ART^4^ (%)** | **N (%) with optimal monitoring to 12m^5^** | **N (%) with optimal monitoring to 24m^6^** |
| --- | --- | --- | --- | --- | --- | --- |
| Clinic 1 | 1515 | 575 / 1313 (43.8%) | 459 / 1245 (36.9%) | 290 / 979 (29.6%) | 120 (9.6 %) | 36 (3.7 %) |
| Clinic 2 | 1354 | 564 / 1117 (50.5%) | 531 / 1060 (50.1%) | 265 / 854 (31.0%) | 280 (26.4%) | 45 (5.3 %) |
| Clinic 3 | 1060 | 318 / 904 (35.2%) | 266 / 844 (31.5%) | 150 / 658 (22.8%) | 80 (9.5 %) | 10 (1.5 %) |
| Clinic 4 | 803 | 145 / 681 (21.3%) | 228 / 648 (35.2%) | 105 / 472 (22.2%) | 39 (6.0 %) | 6 (1.3 %) |
| Clinic 5 | 239 | 92 / 206 (44.7%) | 96 / 193 (49.7%) | 57 / 154 (37.0%) | 40 (20.7%) | 11 (7.1 %) |
| Clinic 6 | 1072 | 231 / 898 (25.7%) | 288 / 850 (33.9%) | 130 / 623 (20.9%) | 33 (3.9 %) | 3 (0.5 %) |
| Clinic 7 | 627 | 278 / 489 (56.9%) | 190 / 463 (41.0%) | 114 / 349 (32.7%) | 94 (20.3%) | 29 (8.3 %) |
| Clinic 8 | 5157 | 1619 / 4141 (39.1%) | 1269 / 3918 (32.4%) | 717 / 3035 (23.6%) | 445 (11.4%) | 192 (6.3 %) |
| Clinic 9 | 681 | 340 / 577 (58.9%) | 125 / 554 (22.6%) | 83 / 438 (18.9%) | 46 (8.3 %) | 12 (2.7 %) |
| Clinic 10 | 1102 | 311 / 921 (33.8%) | 252 / 870 (29.0%) | 121 / 666 (18.2%) | 60 (6.9 %) | 3 (0.5 %) |
| Clinic 11 | 1570 | 653 / 1332 (49.0%) | 422 / 1256 (33.6%) | 267 / 926 (28.8%) | 136 (10.8%) | 23 (2.5 %) |
| Clinic 12 | 3851 | 1233 / 3474 (35.5%) | 866 / 3298 (26.3%) | 475 / 2386 (19.9%) | 176 (5.3 %) | 33 (1.4 %) |
| Clinic 13 | 1871 | 460 / 1542 (29.8%) | 362 / 1449 (25.0%) | 245 / 1117 (21.9%) | 39 (2.7 %) | 6 (0.5 %) |
| Clinic 14 | 493 | 245 / 396 (61.9%) | 204 / 375 (54.4%) | 142 / 308 (46.1%) | 127 (33.9%) | 55 (17.9%) |
| Clinic 15 | 2084 | 982 / 1755 (56.0%) | 853 / 1669 (51.1%) | 415 / 1282 (32.4%) | 482 (28.9%) | 169 (13.2%) |
| Clinic 16 | 1779 | 809 / 1537 (52.6%) | 664 / 1467 (45.3%) | 404 / 1097 (36.8%) | 355 (24.2%) | 114 (10.4%) |
| Clinic 17 | 1113 | 408 / 917 (44.5%) | 310 / 851 (36.4%) | 150 / 355 (42.3%) | 82 (9.6 %) | 16 (4.5 %) |
| **Total** | **26,371** | **9263/22,200 (41.7%)** | **7385/21,010 (35.1%)** | **4130/15,699 (26.3%)** | **2634 (12.5%)** | **1024 (6.5 %)** |

^1^Excludes 3013 patients initiated on ART at the hospital rather than clinics. ^2^Viral load measurement done between >20 weeks to 9 months after ART start. Denominator is individuals who have been on ART at least 9m. ^3^Viral load measurement done between >9 months to 15 months after ART start. Denominator is individuals who have been on ART at least 15m. ^4^Viral load measurement done between >21 months to 27 months after ART start. Denominator is individuals who have been on ART at least 27m. Excludes individuals who started ART in 2016. ^5^Optimal monitoring during first 12m is defined as a viral load measurement at 6 months and 12 months after ART initiation, using the windows in footnotes 1 and 2. Denominator is individuals who have been on ART at least 15 months. ^6^Optimal monitoring during the first 24m is defined as a viral load measurement at 6m, 12m and 24m after ART initiation, using windows in footnotes 2, 3 and 4. Denominator is individuals who have been on ART at least 27 months.

**Supplementary table 2. Proportion of individuals aged ≥15 years with viral load monitoring, and with optimal monitoring, during first 12m and 24m on ART, among those who have been continuously in care without interruption**

| **Year of ART initiation** | **N starting ART** | **N continuously in care for 12m (% of those started) ^1^** | **N continuously in care for 24 months (% of those started) ^1^** | **n with VL at 12m^2^ (%)** | **n with VL at 24m^3^ (%)** | **N (%) with optimal monitoring to 12m^4^** | **N (%) with optimal monitoring to 24m^4^** |
| --- | --- | --- | --- | --- | --- | --- | --- |
| 2010 | 3177 | 2090 (65.8%) | 1812 (57.0%) | 811 (38.8%) | 751 (41.4%) | 341 (16.3%) | 203 (11.2%) |
| 2011 | 3543 | 2517 (71.0%) | 2089 (59.0%) | 955 (37.9%) | 678 (32.5%) | 358 (14.2%) | 142 (6.8 %) |
| 2012 | 4040 | 2765 (68.4%) | 2283 (56.5%) | 897 (32.4%) | 722 (31.6%) | 314 (11.4%) | 135 (5.9 %) |
| 2013 | 3642 | 1955 (53.7%) | 1478 (40.6%) | 672 (34.4%) | 546 (36.9%) | 194 (9.9 %) | 87 (5.9 %) |
| 2014 | 3988 | 2091 (52.4%) | 1487 (37.3%) | 822 (39.3%) | 408 (27.4%) | 211 (10.1%) | 61 (4.1 %) |
| 2015 | 5556 | 3053 (54.9%) | 2095 (37.7%) | 1062 (34.8%) | 289 (13.8%) | 429 (14.1%) | 65 (3.1 %) |
| 2016 | 5438 | 2842 (52.3%) | - | 1307 (46.0%) | - | 568 (20.0%) | - |
| **Total** | **29,384** | **17,313 (58.9%)** | **11,244 (38.3%)** | **6526 (37.7%)** | **3394 (30.2%)** | **2415 (13.9%)** | **693 (6.2 %)** |

^1^Individuals who have been attending clinic at all scheduled visits for at least 15m, or at least 27m (to allow for +3m window). ^2^Viral load measurement done between >9 months to 15 months after ART start. Denominator is individuals who have been continuously in care for at least 15m. ^3^Viral load measurement done between >21 months to 27 months after ART start. Denominator is individuals who have been on ART at least 27m. ^4^Optimal monitoring during first 12m is defined as a viral load measurement at 6 months and 12 months after ART initiation, using a + / -3m window at each time-point. Denominator is individuals who have been on ART at least 15 months. ^5^Optimal monitoring during the first 24m is defined as a viral load measurement at 6m, 12m and 24m after ART initiation, using a + / -3m window at each time-point. Denominator is individuals who have been on ART at least 27 months.

**Supplementary Table 3. Proportion of individuals aged ≥15 years starting ART in each year, and still in care at 6, 12 and 24 months**

| **Year of ART initiation** | **N starting ART** | **N still in care at 6m  (%) ^1^** | **N still in care at 12m (%) ^1^** | **N still in care at 24 m (%) ^1^** |
| --- | --- | --- | --- | --- |
| 2010 | 3177 | 2495 (78.5%) | 2354 (74.1%) | 2150 (67.7%) |
| 2011 | 3543 | 2961 (83.6%) | 2823 (79.7%) | 2597 (73.3%) |
| 2012 | 4040 | 3406 (84.3%) | 3235 (80.1%) | 2993 (74.1%) |
| 2013 | 3642 | 2897 (79.5%) | 2741 (75.3%) | 2523 (69.3%) |
| 2014 | 3988 | 3231 (81.0%) | 3045 (76.4%) | 2740 (68.7%) |
| 2015 | 5556 | 4695 (84.5%) | 4428 (79.7%) | 3962 (71.3%) |
| 2016 | 5438 | 4514 (83.0%) | 4181 (76.9%) | - |
| **Total** | **29,384** | **24,199 (82.4%)** | **22,807 (77.6%)** | **16,965 (70.8%) ^2^** |

^1^Individuals who are attending the clinic (with or without interruptions in care) for at least 9m, 15m or 27m. ^2^Among 23,946 individuals starting ART from 2010-2015

**Supplementary Table 4. Individuals aged ≥15 years with viral load ≥1000, and proportion with repeat test in 6 or 12 months, among individuals starting ART in 2010-2016**

| **Year of ART initiation** | **N with any VL test^1^** | **N (%) with VL ≥1000^2^** | **N (%) with repeat test in 6m^3^** | **N (%) with VL ≥1000 on repeat test^4^** | **N (%) switched to second line^5^** | **Median (IQR) days to switch** | **Switched or VL <1000 on repeat test^4^** |
| --- | --- | --- | --- | --- | --- | --- | --- |
| 2010 | 2328 | 433 (18.6%) | 142 (32.8%) | 96 (67.6%) | 50 (52.1%) | 526 (246-1004) | 96 (67.6%) |
| 2011 | 2754 | 398 (14.5%) | 121 (30.4%) | 78 (64.5%) | 38 (48.7%) | 221.5 (100-567) | 81 (66.9%) |
| 2012 | 3037 | 383 (12.6%) | 102 (26.6%) | 68 (66.7%) | 24 (35.3%) | 247.5 (99.5-500.5) | 58 (56.9%) |
| 2013 | 2279 | 264 (11.6%) | 56 (21.2%) | 29 (51.8%) | 6 (20.7%) | 148.5 (93-963) | 33 (58.9%) |
| 2014 | 2298 | 231 (10.1%) | 76 (32.9%) | 43 (56.6%) | 12 (27.9%) | 520 (204-709.5) | 45 (59.2%) |
| 2015 | 3334 | 201 (6.0 %) | 74 (36.8%) | 41 (55.4%) | 8 (19.5%) | 102.5 (43.5-324.5) | 41 (55.4%) |
| 2016 | 3552 | 225 (6.3 %) | 87 (38.7%) | 53 (60.9%) | 3 (5.7 %) | 338 (283-363) | 37 (42.5%) |
| **Total** | **19,582** | **2135 (10.9%)** | **658 (30.8%)** | **408 (62.0%)** | **141 (34.6%)** | **338 (135-713)** | **391 (59.4%)** |

^1^Viral load measurement done at any point >20 weeks after ART start. ^2^Among individuals with at least one VL test >20 weeks after ART start. ^3^Repeat viral load done between 10-30 weeks after VL ≥1000, among those with VL measurement ≥1000. **^4^**Among those with repeat test in 10-30 weeks. **^5^**Among those with repeat VL ≥1000.

**Supplementary Table 5. Factors associated with success in the first line cascade, among 2135 individuals aged ≥15 years with a viral load ≥1000 copies/mL**

|  | n success / N(%)^1^ | Crude odds ratio  (95% CI) | Adjusted odds ratio (95%CI) ^2^ |
| --- | --- | --- | --- |
| Age at VL ≥1000^3^ |  | P=0.39^4^ | P=0.14^4^ |
| 15-24 years | 78 / 365 (21.4%) | 1.22 (0.91 -1.62 ) | 1.34 (0.99 -1.83 ) |
| 25-34 years | 162 / 937 (17.3%) | 1 | 1 |
| 35-44 years | 87 / 534 (16.3%) | 0.89 (0.73 -1.07 ) | 0.84 (0.68 -1.03 ) |
| ≥45 years | 64 / 299 (21.4%) | 0.94 (0.75 -1.17 ) | 0.91 (0.71 -1.16 ) |
| Sex |  | P=0.008 | P=0.11 |
| Male | 116 / 757 (15.3%) | 1 | 1 |
| Female | 275 / 1378 (20.0%) | 1.38 (1.09 -1.75 ) | 1.23 (0.95 -1.60 ) |
| Duration on ART^3^ |  | P<0.001^5^ | P<0.001^5^ |
| < 2 years | 17 / 299 (5.7 %) | 0.77 (0.66 -0.90 ) | 0.78 (0.67 -0.90 ) |
| 2 - <5 years | 141 / 876 (16.1%) | 1 | 1 |
| 5 - <7 years | 92 / 489 (18.8%) | 2.05 (1.53 -2.76 ) | 2.05 (1.52 -2.75 ) |
| ≥7 years | 141 / 471 (29.9%) | 2.99 (2.25 -3.98 ) | 3.06 (2.28 -4.09 ) |
| ART regimen when failing |  | P=0.59 | P=0.91 |
| TDF, FTC, EFV | 194 / 1131 (17.2%) | 1 | 1 |
| TDF, EFV, 3TC | 114 / 601 (19.0%) | 1.13 (0.88 -1.46 ) | 0.87 (0.66 -1.14 ) |
| ABC, EFV, 3TC | 3 / 24 (12.5%) | 0.69 (0.20 -2.34 ) | 0.67 (0.19 -2.34 ) |
| AZT, EFV, 3TC | 6 / 30 (20.0%) | 1.21 (0.49 -2.99 ) | 1.07 (0.42 -2.74 ) |
| Other | 3 / 14 (21.4%) | 1.32 (0.36 -4.77 ) | 1.05 (0.28 -3.92 ) |
| Unknown | 71 / 335 (21.2%) | 1.30 (0.96 -1.76 ) | 0.99 (0.71 -1.37 ) |

^1^Success defined as repeat measurement within 6 months, and viral load <1000 on repeat testing, or switch to appropriate second line therapy. Denominator is all individuals with a viral load ≥1000. ^2^Adjusted for age, sex, duration on ART and ART regimen when failing. ^3^Continuous covariates (age and duration on ART) modelled by restricted cubic splines with 3 knots; n (%) success in each age group shown for information only. ^4^Odds ratios are presented at selected values of age, comparing the minimum value in each age group to age 25 as the reference. ^5^Odds ratios presented at selected values of time on ART, comparing the minimum value in each group to 2 years as the reference.
